# Emerging Insights on the Diverse Roles of Proprotein Convertase Subtilisin/Kexin Type 9 (PCSK9) in Chronic Liver Diseases: Cholesterol Metabolism and Beyond

**DOI:** 10.3390/ijms23031070

**Published:** 2022-01-19

**Authors:** Thomas Grewal, Christa Buechler

**Affiliations:** 1 School of Pharmacy, Faculty of Medicine and Health, University of Sydney, Sydney, NSW 2006, Australia; thomas.grewal@sydney.edu.au; 2 Department of Internal Medicine I, Regensburg University Hospital, 93053 Regensburg, Germany

**Keywords:** PCSK9, LDL-receptor, hepatitis C, NAFLD, alcoholic liver disease, visceral obesity

## Abstract

Chronic liver diseases are commonly associated with dysregulated cholesterol metabolism. Proprotein convertase subtilisin/kexin type 9 (PCSK9) is a serine protease of the proprotein convertase family that is mainly synthetized and secreted by the liver, and represents one of the key regulators of circulating low-density lipoprotein (LDL) cholesterol levels. Its ability to bind and induce LDL-receptor degradation, in particular in the liver, increases circulating LDL-cholesterol levels in the blood. Hence, inhibition of PCSK9 has become a very potent tool for the treatment of hypercholesterolemia. Besides PCSK9 limiting entry of LDL-derived cholesterol, affecting multiple cholesterol-related functions in cells, more recent studies have associated PCSK9 with various other cellular processes, including inflammation, fatty acid metabolism, cancerogenesis and visceral adiposity. It is increasingly becoming evident that additional roles for PCSK9 beyond cholesterol homeostasis are crucial for liver physiology in health and disease, often contributing to pathophysiology. This review will summarize studies analyzing circulating and hepatic PCSK9 levels in patients with chronic liver diseases. The factors affecting PCSK9 levels in the circulation and in hepatocytes, clinically relevant studies and the pathophysiological role of PCSK9 in chronic liver injury are discussed.

## 1. Introduction

Proprotein convertase subtilisin/kexins (PCSKs) are serine proteases that convert inactive proproteins into their active forms by proteolysis. These proteins undergo autocatalytic cleavage of the N-terminal prodomain, which noncovalently binds to the catalytic domain. Activation of PCSKs requires cleavage of the prodomain, which then dissociates from the complex. However, the mode of action of PCSK9 is strikingly different to other PCSK family members, as the PCSK9 prodomain does not detach from the catalytic domain after cleavage, only to remain noncovalently attached to the mature and secreted PCSK9 protein. This dimer, which does not possess protease activity, represents the biologically active PCSK9 variant [1,2].

**Figure 1:**
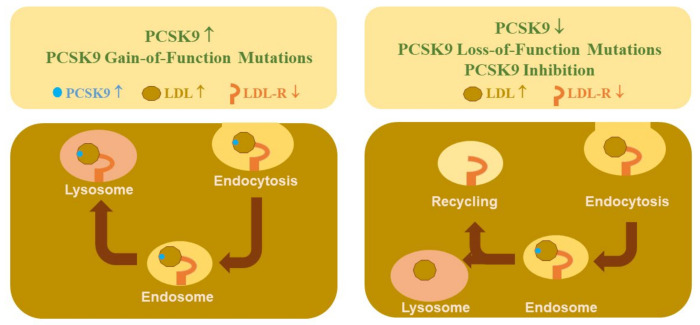
Regulation of LDL and hepatic LDL-R levels by PCSK9. LDL bound to the LDL-R is taken up by endocytosis, LDL is degraded in the lysosome and LDL-R is recycled. Binding of PCSK9 to the LDL–LDL-R complex induces lysosomal degradation of the LDL-R. High PCSK9 levels and PCSK9 gain-of-function mutations favor LDL-R degradation leading to low hepatic LDL-R protein and high LDL levels in plasma. Low PCSK9 levels, loss-of-function mutations and PCSK9 blockage favor LDL-R recycling and lead to higher abundance of the hepatic LDL-R and improved clearance of circulating LDL.

Almost two decades ago, several studies identified a role for PCSK9 in the regulation of hepatic low-density lipoprotein-receptor (LDL-R) protein levels and a link to hypercholesterolemia. One of these studies recognized PCSK9 as a dietary cholesterol-responsive gene in the liver of mice. PCSK9 was also found in screening assays for hepatic genes regulated by sterol regulatory element-binding proteins (SREBPs), transcription factors that control lipid homeostasis [3,4,5]. Soon thereafter, the central role for PCSK9 in hepatic cholesterol uptake was unraveled, identifying PCSK9 to increase serum LDL levels by targeting the LDL-R for lysosomal degradation [6,7] (Figure 1). Moreover, critically validating the potential of targeting PCSK9 in hypercholesterolemic patients, gain- and loss-of-function PCSK9 mutations in humans were associated with high and low serum cholesterol levels, respectively [6,8,9,10,11] (Figure 1).

Over the years, other receptors that also bind PCSK9 have been identified, including CD36 and the very-low-density lipoprotein receptor (VLDL-R), with consequences for fatty acid and triglyceride metabolism [12] (see below).

Human PCSK9 circulates as a mature form (~62 kDa) as well as an N-terminally truncated form (~55 kDa) [13]. PCSK9 truncation is mediated by furin, another proprotein convertase produced by hepatocytes and representing the main PCSK9 processing enzyme in vivo. The furin cleavage site within PCSK9 is RFHR^218^↓, where PCSK9 is cleaved after the arginine residue at position 218 at the end of the furin consensus sequence Arg-Phe-His-Arg, and up to now, three gain-of-function mutations (R215H, F216L, R218S), have been found within this sequence [9,10,14]. These three mutations impair cleavage of PCSK9 by furin [15,16], increasing the ability of PCSK9 to downregulate LDL-R and elevate serum cholesterol levels. In line with these findings, the shortened PCSK9 form did not alter LDL-R degradation kinetics [13]. Yet, other studies described that furin-cleaved PCSK9 can still induce LDL-R degradation, though with a ~2-fold lower efficiency than full-length PCSK9 [17,18]. Hence, the furin-dependent turnover of PCSK9 in plasma is critical for cholesterol homeostasis, and appears to be tightly regulated. Indeed, the shorter PCSK9 isoform constitutes to 15–40% of the total PCSK9 levels in human plasma, and 30–50% in murine plasma [13,15,16].

Despite the clinical relevance of determining the ratio of long and short circulating PCSK9 isoforms, this information is often not readily available when using commercially available ELISAs, as microplates coated with monoclonal PCSK9 antibodies may not react with similar affinity to both PCSK9 isoforms. Thus, the comparison of absolute and/or truncated PCSK9 levels in studies that used different ELISAs to determine circulating PCSK9 isoforms is still problematic. As such, PCSK9 concentrations varied widely between various patient cohorts and ranged from 50–600 ng/mL [19,20,21,22,23,24].

Potentially further contributing to the substantial variations observed in patient cohorts, it appears that serum PCSK9 levels have a diurnal rhythm with the lowest levels later in the day and highest levels in early morning [25]. These findings correspond to the variation of hepatic cholesterol synthesis over a 24 h period [26], indicating that the diurnal regulation of LDL-cholesterol uptake and cholesterol synthesis are coordinated with PCSK9 levels in the circulation, ensuring constant serum LDL concentrations throughout diurnal rhythms [25].

Gender, prolonged fasting and the postprandial state are also associated with changes of circulating PCSK9 levels. For instance, fasting (>18 h), but also immediately after feeding, led to a decline in PCSK9 plasma levels [25,27]. In addition, most studies have shown that plasma PCSK9 concentrations are higher in females than males [7,28,29].

Although circulating PCSK9 is mostly derived from the liver [16], the ability of PCSK9 to bind and promote hepatic LDL-R degradation also implicates that the liver is involved in the clearance of PCSK9 [30]. Along these lines, comparable concentrations of PCSK9 in the hepatic and portal vein of patients with liver cirrhosis suggest that the amounts of PCSK9 secreted and cleared by the liver are similar [31], an observation that has yet to be confirmed in healthy-liver patients. Remarkably, the half-life of PCSK9 is increased 3-fold in LDL-R knock-out (KO) mice [30], indicating that besides the LDL-R, other molecules and receptors contribute to the clearance of PCSK9. Adding further complexity to the regulation of PCSK9 levels in the circulation, some evidence indicates that PCSK9 is also excreted by the kidney [12].

Two anti-PCSK9 monoclonal antibodies, evolocumab and alirocumab, were recently approved for clinical use within the European Union [7]. These antibodies block the binding of PCSK9 to the LDL-R, thereby inhibiting PCSK9-mediated stimulation of LDL-R degradation. Consequently, increased LDL-R levels at the cell surface bind and internalize elevated numbers of LDL particles, ultimately lowering LDL-cholesterol levels in the circulation. Therapies using PCSK9 inhibitors are recommended for patients with a high risk for cardiovascular events, in particular those that do not tolerate or do not properly respond to conventional lipid-lowering drugs [32]. Evolocumab and alirocumab therapies, either alone or in combination with statins, reduce LDL-cholesterol by 60% [33]. Two large-scale clinical trials did not report on serious adverse events over a median follow-up of 2.2 and 2.8 years, respectively [33,34]. However, further analysis revealed that ~20% of patients experienced insomnia, headache and depression upon PCSK9 inhibitor therapy, pointing at the need for further clinical data to be collected [34].

Monoclonal antibodies have to be injected and PCSK9 blocking drugs appropriate for oral administration are being developed [35].

Despite the approval of PCSK9 inhibitors in combination with statins, their mode of action when taken together is still not fully understood. Statins inhibit 3-hydroxy-3-methylglutaryl-coenzyme A (HMG-CoA) reductase, and thereby block cholesterol biosynthesis. This leads to the activation of SREBPs and transcriptional upregulation of LDL-R expression. However, statin-mediated SREBP activation also increases hepatic PCSK9 expression, causing systemic PCSK9 levels to rise by 6–39% during statin therapy [8]. Likewise, use of statins was associated with approximately 14% higher PCSK9 levels in bacteraemia patients [36]. Thus, the percentage of patients taking statins has to be considered when comparing PCSK9 levels and treatment efficacy between different cohorts.

Ezetimibe is another approved cholesterol-lowering drug that inhibits Niemann–Pick C1-like protein 1 (NPC1L1), a transmembrane protein enriched in the apical membrane of enterocytes in the small intestine, mediating extracellular sterol transport across the brush border membrane. NPC1L1 is essential for intestinal sterol absorption, and its inhibition using ezetimibe effectively reduces uptake of dietary cholesterol [33]. In addition, ezetimibe therapy also blocks hepatic NPC1L1, upregulates hepatic LDL-R levels and stimulates biliary cholesterol excretion [37,38]. In contrast to statin-induced hepatic PCSK9 expression, there is little evidence that ezetimibe monotherapy impacts PCSK9 levels or activity or has an additional effect on circulating PCSK9 levels when added to statin therapy [8].

Interestingly, other lipid-lowering drugs not directly targeting elevated serum cholesterol levels also influence circulating PCSK9 amounts. This includes fibrates, which are commonly used to lower triglycerides via activation of the transcription factor peroxisome proliferator-activated receptor (PPAR) alpha. This member of the nuclear receptor superfamily is predominantly expressed in the liver. Fibrates not only stimulate PPARalpha-inducible gene expression patterns with key roles in hepatic lipid metabolism, including fatty acid beta-oxidation, but also downregulate PCSK9 expression in hepatocytes [39,40]. Given that hepatic PCSK9 secretion is the main determinant of PCSK9 levels in plasma and that fenofibrate therapy of patients effectively reduced circulating PCSK9 levels [40], one can speculate that fibrate-stimulated and PPARalpha-mediated PCSK9 downregulation in hepatocytes is the underlying cause for fibrates lowering circulating amounts of PCSK9.

Besides the impact of the various lipid-lowering drugs on PCSK9 expression and activity in patients, endogenous regulators of PCSK9 in plasma appear to exist. For instance, a member of the annexin protein family, annexin A2 (AnxA2), with a plethora of functions in vascular homeostasis [41], binds to PCSK9 on the cell surface, thereby inhibiting PCSK9-mediated LDL-R degradation [42,43]. This might be particularly relevant for PCSK9 Q554E mutant carriers, as this mutation displayed increased binding affinity towards AnxA2 and strongly diminished ability to promote LDL-R degradation [42]. Furthermore, AnxA2 knock-out mice exhibited elevated LDL-cholesterol and circulating PCSK9 levels, while LDL-R levels were reduced in extrahepatic tissues. AnxA2 is only moderately expressed in the liver, but adenoviral AnxA2 overexpression elevated hepatic LDL-R levels [43]. The association of PCSK9 and LDL-cholesterol levels with AnxA2 gene variants [43,44] indicates that single nucleotide polymorphisms and mutations in the AnxA2 gene and other yet unidentified PCSK9 modulators need to be considered when assessing the efficacy of drugs aiming to block PCSK9-mediated LDL-R degradation to lower LDL-cholesterol levels.

It is important to note that the development of chronic liver diseases does not only originate from liver dysfunction, but often reflects deregulated whole-body physiology driven by other tissues that communicate with the liver. In this context, the liver is closely connected to the intestine through the portal vein. As metabolites from visceral fat are released into the portal vein, visceral adiposity worsens most, if not all, chronic liver diseases. In fact, metabolites, cytokines, chemokines and adipokines produced by the gut or adipose tissue contribute to liver steatosis, inflammation and fibrosis [45,46,47,48]. Thus, the expression and the function of PCSK9 in the intestine, adipose tissues and its association with metabolites released by these organs critically influences liver function and will also be described in this review article.

## 2. Roles for PCSK9 in the Intestine

There is a close association between the gut and the liver, which is affected by genetic and environmental factors. Disruption of the gut–liver axis contributes to hepatic dysfunction and chronic liver diseases [49].

Besides the prominent expression of PCSK9 in the liver, PCSK9 is also found in the intestine, and therefore, was considered in postprandial hypertriglyceridemia [50,51]. In rodents, PCSK9 blockage by alirocumab indeed improved postprandial lipemia, while hepatic PCSK9 overexpression had the opposite effect [51]. Plasma lipids were, however, not changed in mice with an intestine-specific loss of PCSK9 [51]. Thus, hepatic rather than intestinal PCSK9 seems to enhance the synthesis of intestinal triglyceride-rich apolipoprotein B48-containing particles, the so-called chylomicrons [50,51]. Despite some intestinal PCSK9 synthesis, this does not significantly contribute to plasma levels of PCSK9 in mice [51], and further analysis is needed to clarify the function of PCSK9 in the intestine in humans.

In relation to PCSK9 modulating cholesterol homeostasis in the intestine, administration of purified recombinant PCSK9 to the intestinal cell line Caco-2/15 enhanced the synthesis of cholesterol, which consequently decreased LDL-R protein levels. Interestingly, this induction of the well-established feedback-loop in cholesterol homeostasis was accompanied by an elevated release of chylomicrons [52]. Indicating further roles for PCSK9 in intestinal cells, addition of a recombinant gain-of-function PCSK9 protein even induced cholesterol uptake and expression of sterol (NPC1L1) and fatty acid (CD36) transporters in these cells [52].

In humans, the inhibition of PCSK9 with evolocumab only modestly lowered intestinal cholesterol absorption [53]. This therapeutic antibody had no effect on the metabolism of apolipoprotein B-48 in response to an oral fat load. On the other hand, catabolism of these apolipoprotein B-48 containing chylomicron particles was enhanced by atorvastatin, and this was observed in the fasting and the postprandial state [54]. This study enrolled healthy, normolipidemic men [54] and it has yet to be determined if the effect of PCSK9 blockage on intestinal apolipoprotein B-48 and triglyceride metabolism may be more pronounced in hypercholesterolemic patients with higher circulating PCSK9 levels. Indeed, individuals with PCSK9 loss-of-function mutations had lower fasting and postprandial levels of triglycerides, apolipoprotein B-48 and total apolipoprotein B levels [27], suggesting a function of PCSK9 in chylomicron production.

## 3. Circulating PCSK9 and LDL-Cholesterol Levels in Different Patient Cohorts

As outlined above, the role of PCSK9 in the hepatic clearance of LDL-cholesterol is now well-established, culminating in approved therapies that effectively lower LDL-cholesterol levels, in particular in hypercholesterolemic patients that respond poorly to other cholesterol-lowering drugs [6,12,55]. However, the liver not only controls cholesterol homeostasis, but communicates with other organs to coordinate overall lipid homeostasis in the body. Moreover, lipid metabolism is commonly disturbed in diabetic and obese patients during persistent and acute inflammation as well as in patients with chronic liver diseases [56,57,58,59,60,61,62], and often not directly related to defects in LDL-cholesterol uptake. Strikingly, increasing evidence points at additional roles for PCSK9 in the liver beyond cholesterol uptake via the LDL-R. In the following, we will summarize studies that have analyzed PCSK9 levels and lipoproteins in patients and experimental models suffering from metabolic disorders, including diabetes, obesity as well as inflammation and chronic liver injury.

In healthy individuals, various studies have shown that circulating PCSK9 levels positively correlate with serum LDL-cholesterol and total cholesterol, but not with triglycerides or high-density lipoprotein (HDL)-cholesterol concentrations [8,63,64]. Such associations were also observed in a study of 115 diabetic patients [40], yet another cohort of 267 patients with metabolic syndrome or type 2 diabetes displayed plasma PCSK9 levels that correlated positively not only with total cholesterol, but also with triglycerides and apolipoprotein B. Large VLDL, intermediate-density lipoprotein, small LDL and HDL particles and cholesterol in remnant lipoproteins are all atherogenic lipoproteins, and were positively associated with plasma PCSK9 levels [65]. Serum PCSK9 concentrations also correlated with atherogenic lipoproteins (lipoprotein (a), small-dense LDL and oxidized LDL) in patients with coronary artery disease [64]. It can be speculated that these positive associations of circulating PCSK9 amounts with various atherogenic lipoprotein levels of different patient cohorts are likely due to the key functions of PCSK9 in cholesterol homeostasis.

Notably, not all cohort studies could identify a positive correlation between circulating PCSK9 and LDL-cholesterol levels [66], an observation that could possibly be explained by medication use. In fact, recent studies documented 23% and 9% of the US and German population, respectively, being on statin therapy [67,68]. As described above, while statins lower LDL-cholesterol, these drugs also increase hepatic PCSK9 expression [8], which may interfere with the positive association of PCSK9 and LDL-cholesterol levels observed in some cohorts.

In addition, impaired lipid metabolism in patients with chronic liver diseases also needs to be considered. For instance, low LDL-cholesterol levels are common in patients with liver cirrhosis or hepatitis C [69,70]. Thus, besides lipid-lowering medications, underlying illnesses may also affect circulating cholesterol levels in a manner unrelated to PCSK9 plasma concentrations. Remarkably, although individuals carrying PCSK9 loss-of-function mutations have a lower risk for cardiovascular events [11,71,72], LDL-cholesterol was only reduced by 10% in humans carrying the R46L variant [73]. Hence, loss of PCSK9 function may confer cardiovascular protection even without a significant reduction in circulating LDL-cholesterol levels [11,71,72]. In these PCSK9 R46L mutant carriers, several cholesteryl ester and sphingolipid species in the plasma were decreased, indicating that reduced levels of low abundant, biologically active lipid species in R46L variants may contribute to cardioprotection [73]. It remains to be determined if these lower amounts of specific lipid species in R46L carriers are associated with the lipid composition of LDL particles, which carry about 60% of serum ceramides and 30–50% of serum sphingomyelin [74,75]. Likewise, PCSK9 inhibition in patients lowered sphingolipid levels in plasma. Additionally, administration of monoclonal PCSK9 antibodies increased HDL phospholipid content [76], which may lead to a higher cholesterol efflux capacity and improve the ability of HDL to protect from cardiovascular diseases [77]. Thus, current data suggest beneficial effects of therapeutic anti-PCSK9 antibodies beyond lowering LDL cholesterol. However, it is evident that further comparison of the lipidome from isolated lipoproteins of healthy individuals, PCSK9 mutant carriers and patient cohorts treated with or without PCSK9 inhibitors, is needed to identify all clinically relevant changes in lipid profiles linked to PCSK9 blockage.

## 4. Regulation of Hepatocyte PCSK9 by Cytokines and Adipokines

Obesity is an independent risk factor for chronic liver diseases such as alcoholic liver disease, hepatitis C virus (HCV) infection, nonalcoholic fatty liver disease (NAFLD) and even hepatocellular carcinoma (HCC) [78]. Adipose tissues produce a variety of cytokines and adipokines, whose circulating levels are typically elevated in the obese state [78,79]. The majority of these proteins are known to contribute to the initiation and progression of chronic liver diseases [48,79], and as outlined below, often with consequences for PCSK9 expression and function.

Leptin is a well-described satiety hormone released from adipocytes and commonly upregulated in obesity [78,79], capable of enhancing inflammatory and profibrotic processes in the liver [46]. In the HepG2 hepatocellular carcinoma cell line, which frequently serves as a hepatocyte model, leptin increased the expression of PCSK9, causing reduced LDL-R levels and impaired LDL uptake [80]. The ability of leptin and resistin, another adipokine, to induce PCSK9 expression involves signal transducer and activator of transcription (STAT) 3 [80,81]. STAT3 is also activated by proinflammatory cytokines, which are often upregulated in hyperleptinemia [82]. In this context, the proinflammatory tumor necrosis factor (TNF), through the activation of the suppressor of cytokine signaling (SOCS) 3 protein, stimulated the STAT3-dependent pathway to increase PCSK9 expression [83].

Interleukin-6 (IL-6) and oncostatin M, another member of the IL-6 family, both cytokines with established links to obesity and inflammatory disorders, are also potent activators of STAT3, and contribute to acute and chronic liver pathophysiology [84,85]. Oncostatin M is an effective inducer of liver fibrosis [86] and was shown to increase LDL-R levels in cells and hypercholesterolemic rabbits, possibly via inhibition of PCSK9. However, the repressive effect of oncostatin M on PCSK9 expression appeared to be unrelated to STAT3, but rather triggered through janus kinase (JAK) and extracellular signal-regulated kinase (ERK) signaling networks [84].

IL-6 is a pleiotropic cytokine with various functions such as the induction of the acute phase response in the liver after infection, inflammation, or injury [85]. Yet, despite the ability of IL-6 to activate a variety of transcription factors, including STAT3, PCSK9 levels remained unchanged in the presence of IL-6 [87]. Notably, anti-IL-6 therapy with tocilizumab of patients with an acute coronary syndrome also did not change serum PCSK9 levels [88]. In addition, several other immunoregulatory cytokines, including IL-18, TNF, interferon (IFN) gamma and IFNalpha did not alter PCSK9 protein levels, while IL-1beta caused a 50% reduction in PCSK9 expression [87]. It should be noted that in vivo evidence for these observations is still lacking, as the majority of these experiments were performed using the HepG2 hepatocellular carcinoma cell line. Furthermore, different study designs may contribute to variable outcomes in cell-based assays when assessing the influence of cytokines (e.g., TNF) on PCSK9 levels [83,87]. Thus, further research, ideally using primary hepatocytes or in vivo models, will have to clarify whether the abovementioned cytokines have any impact on hepatocyte PCSK9 production.

Adiponectin is an exceptional adipokine known for its antidiabetic, anti-inflammatory, antiatherogenic, and cardioprotective effects. Circulating adiponectin levels are decreased in obesity, diabetes, the metabolic syndrome, and atherosclerosis, and there is now convincing evidence for adiponectin to exert multiple protective effects in metabolic liver diseases [79]. Adiponectin binds adiponectin receptors on hepatocytes and other cells, stimulating signaling pathways that regulate many physiological processes, including lipid metabolism. Adiponectin receptor agonists have been developed, and in HepG2 cells and mouse liver, activated the transcription factors PPARgamma and SREBP2, leading to a simultaneous increase in PCSK9 and LDL-R levels [89]. In line with elevated hepatic LDL-R expression in adiponectin receptor agonist-treated mice, total- and LDL-cholesterol levels in serum declined. On the other hand, in apolipoprotein E knock-out mice on a high-fat diet, adiponectin receptor agonists reduced hepatic PCSK9 expression whereas LDL-R levels were increased. In this model, serum levels of LDL-cholesterol were reduced while HDL-cholesterol levels were elevated. The mechanisms responsible for the opposite regulation of PCSK9 by adiponectin receptor agonists in wild-type and apolipoprotein E null mice still have to be clarified [89].

Besides addressing the impact of adipokines on hepatic PCSK9 levels in various disease settings, researchers have also examined if hepatic PCSK9 expression is modulated by inflammatory mediators, such as exposure to lipopolysaccharide (LPS) after bacterial infection. Although LPS did not enhance PCSK9 protein levels in HepG2 cells [87], injection of LPS increased hepatic PCSK9 mRNA expression in mice [90]. This suggests that the positive association of circulating PCSK9 levels with the appearance of key inflammatory markers, such as high-sensitive C-reactive protein or increased white blood cell count, are in part related to the enhanced hepatic PCSK9 production induced by inflammatory factors such as LPS [91].

## 5. PCSK9 and Inflammation

Lipoprotein particles not only transport lipids, but are associated with many other biomolecules. This includes LDL and HDL, which can bind and neutralize bacterial antigens such as LPS [92]. Given the ability of PCSK9 to bind LDL-R and modulate LDL clearance from plasma, PCSK9 was hypothesized to play a role in infectious diseases and inflammation. This was indeed supported by animal studies, as PCSK9 null mice were protected from LPS-induced inflammation in an LDL-R-dependent manner [92]. Likewise, PCSK9 inhibition lowered nuclear factor kappa B (NF-κB) activation and inflammation in an experimental colitis model [93]. Similarly, in a mouse model for sepsis, administration of PCSK9 inhibitor diminished inflammation and increased survival, while PCSK9 overexpression had the opposite effect [94].

Several clinical studies further support a role for PCSK9 in inflammation. Human PCSK9 loss-of-function mutant carriers displayed reduced IL-6 blood levels shortly after LPS injection and were associated with increased survival after sepsis [92]. Sepsis in patients with PCSK9 gain-of-function mutations was related to reduced survival, which correlates with the 2–3-fold increased plasma PCSK9 levels in patients with sepsis and their more fatal illness [95]. In line with these findings, high amounts of PCSK9 impaired hepatocyte clearance of *E. coli* endotoxin [95].

However, it is important to note that not all studies support raised PCSK9 levels to be detrimental in inflammatory diseases. PCSK9 loss-of-function mutations were not linked to a lower risk for infection and sepsis among 10,924 participants enrolled in the REasons for Geographic and Racial Differences in Stroke (REGARDS) cohort [96]. Furthermore, impaired elevation of plasma PCSK9 levels in patients with bacteremia was even associated with the need for more interventions and mortality [36].

While the abovementioned studies examined the potential contribution of PCSK9 to the severity and progression of inflammation, several reports suggest PCSK9 to contribute to the early steps of hepatic inflammation, which is the main driver of liver injury and leads to liver fibrosis and cirrhosis [97]. It is generally believed that antigens derived from the gut and endogenous ligands produced by injured cells activate inflammatory pathways [97], and in the liver, Kupffer cells, infiltrating macrophages, T lymphocytes, neutrophils, and dendritic cells participate in hepatic inflammation [97,98,99]. Although it is not fully clear how PCSK9 secretion in hepatocytes is coordinated in the presence of gut-derived antigens and/or ligands from injured cells, incubation of macrophages with recombinant or HepG2-derived PCSK9 protein led to mRNA upregulation of IL-1beta, IL-6, TNF, and the two chemokines CXCL2 and CCL2 [100]. The fact that PCSK9 secreted from HepG2 cells was much more potent than the recombinant PCSK9 protein to induce inflammatory cytokine expression in macrophages suggests that post-translational modifications in HepG2 cells may render PCSK9 more active. Alternatively, PCSK9 export from cells in this model may be accompanied by the secretion of additional proinflammatory mediators. Though the respective pathways have not been characterized in detail, LDL-R expression on the surface of macrophages appeared critical to exerting the proinflammatory effect of PCSK9 on macrophages [100].

In fact, numerous studies were able to connect low hepatic PCSK9 levels with decreased amounts of proinflammatory factors such as TNF, IL-6, CXCL8, CCL2 and CXCL2. Additionally, blockage of PCSK9 in animal models decreased systemic inflammation [91]. Although these and various other reports suggested PCSK9 to act as an inflammatory factor, PCSK9 depletion in HepG2 cells enhanced TNF and SOCS3 expression [101]. Taken together, while these experiments implicate concentration-dependent roles for PCSK9 in hepatic inflammation, further research is needed to clarify how PCSK9 may act as a pro- or anti-inflammatory molecule.

## 6. PCSK9 in Patients with Liver Cirrhosis and Mixed Disease Etiology

NAFLD, alcohol abuse and infection with hepatitis B virus (HBV) or HCV are major causes for chronic liver diseases. The final stage (end-stage) of chronic liver injury is liver cirrhosis [46], which often triggers HCC. In the context of NAFLD, HCC may also develop in the noncirrhotic liver [102,103]. As the liver is the main organ for lipid metabolism, liver dysfunction due to chronic liver injury is associated with dyslipidemia. In liver cirrhosis, largely preserved liver function is associated with higher circulating cholesterol levels. Notably, low plasma cholesterol concentrations of patients with liver cirrhosis are linked to increased mortality [104].

As compromised liver function in liver cirrhosis has consequences for plasma cholesterol levels, several studies have investigated PCSK9 quantities in end-stage liver disease. The model of end-stage liver disease (MELD) score is an estimate for the survival of patients with end-stage liver disease and is based on serum bilirubin, creatinine, and the international normalized ratio (INR) [46]. In a patient cohort with end-stage liver disease and mixed disease etiology, a negative correlation of serum PCSK9 concentrations with the MELD score was observed and low PCSK9 levels were associated with a higher mortality [24]. Indicating a disconnect between PCSK9 and serum cholesterol levels in chronic liver disease, concentrations of total cholesterol and cholesterol precursors were similar in patients with high and low PCSK9 levels [24].

In patients with liver cirrhosis, serum PCSK9 concentrations were markedly reduced in comparison to healthy controls [24]. Bacteremia patients with liver diseases also had low plasma PCSK9 levels in comparison to bacteremia patients with normal liver function [36]. One can speculate that decreased levels of circulating PCSK9 in liver fibrosis could be due to dysfunctional PCSK9 synthesis in hepatocytes of the damaged liver.

A further study also found that serum PCSK9 levels were 20–30% higher in healthy controls compared to patients with chronic hepatitis or liver cirrhosis [20]. It remains to be determined if serum PCSK9 concentrations decline over time during chronic liver injury to reach lowest levels at end-stage liver disease. It is also unclear if the etiology of liver disease in this cohort (HBV, n = 14; HCV, n = 15; ethanol abuse; n = 3; non-alcoholic steatohepatitis (NASH), n = 4; other etiologies, n = 3) impacts hepatic PCSK9 expression, secretion and/or PCSK9 circulation [20].

In contrast to the trends described above, one study using immunohistochemistry for PCSK9 detection documented increased PCSK9 levels in the cirrhotic liver [20]. However, hepatic PCSK9 protein levels in patients (10 HBV, n = 10; HCV, n = 11; nonviral disease etiology, n = 11) with fibrosis stage 1, 2 or 3 were comparable to patients with liver cirrhosis (fibrosis stage 4) [31], indicating that progression of liver cirrhosis may not be accompanied by a continuous lowering of PCSK9 protein levels in the liver. Despite severely compromised liver function in these patients, hepatic PCSK9 protein expression positively correlated with LDL-R protein levels [31]. Interestingly, PCSK9 protein levels were increased in the liver of HCV patients when compared to patients with nonviral liver disease, indicating that viral infection may contribute to influencing the machinery responsible for PCSK9 expression and secretion [31]. Future research with larger patient cohorts may be able to determine if PCSK9 levels are impacted by different etiologies of liver disease rather than progression of fibrosis.

## 7. PCSK9 and Alcoholic Liver Disease

Excessive and continuously high intake of ethanol causes alcoholic liver disease, which ranges from alcoholic fatty liver to hepatitis, liver fibrosis and cirrhosis [105,106]. Fatty liver is an early stage and develops in ~60–90% of patients [58,107]. Disturbed hepatic lipid metabolism is a key feature of alcoholic liver disease, and liver steatosis is a risk factor for progressive liver damage [105]. Therefore, somewhat unsurprisingly, several features associated with alcoholic liver disease are likely to influence the role of PCSK9 in cholesterol homeostasis and liver function. For example, ethanol enhances hepatic triglyceride and cholesterol synthesis, and hyperlipidemia as well as hypercholesterolemia are common in these patients [58,108]. In addition, ethanol impairs the integrity of the intestinal barrier and bacterial antigens such as LPS can enter the blood [109], which induces the production of proinflammatory cytokines, chemokines, and reactive oxygen species in Kupffer cells and hepatocytes, as described above [109,110,111].

Supporting PCSK9 deregulation upon chronic alcohol exposure, analysis of end-stage alcoholic liver disease patients identified reduced hepatic PCSK9 mRNA levels in comparison to healthy-liver controls [112]. Serum PCSK9 levels were also decreased in these patients when compared to noncirrhotic controls [31]. While this can in part be explained by liver toxicity, genome-wide DNA analysis revealed increased methylation of the PCSK9 promoter in the liver of these patients, which correlated with low PCSK9 expression, indicating that epigenetic regulation of the PCSK9 gene contributes to control the ability of transcription factors to bind and stimulate PCSK9 transcription [112].

Associations of serum PCSK9 levels with circulating markers of liver function such as bilirubin, aminotransferases or the MELD score did not exist in patients with alcoholic cirrhosis [31]. The accumulation of ascites is often an early manifestation of acute deterioration of liver function in cirrhosis patients, also termed decompensated cirrhosis [113], but plasma PCSK9 levels remained unchanged in patients with decompensated disease [31]. In alcoholic cirrhosis patients, plasma PCSK9 amounts did not correlate with C-reactive protein or IL-6 levels, which are commonly regarded as markers of systemic inflammation [31,113].

In contrast to the human studies listed above, a rodent model of alcoholic liver disease was characterized by increased hepatic PCSK9 mRNA levels. Yet, this was not accompanied by LDL-R downregulation, indicating mechanisms that prevent increased PCSK9 translation, secretion or activity. Alirocumab blocked ethanol-mediated PCSK9 upregulation, and moreover, induced hepatic LDL-R protein levels not only in controls, but also ethanol-fed animals [114]. Treatment with PCSK9 inhibitor improved alcoholic hepatic steatosis, reduced hepatic triglyceride levels and ameliorated hepatocyte death and liver inflammation. In this study, one adverse effect was a rise in serum triglycerides and free fatty acids [114], highlighting that further studies investigating the role of PCSK9 in alcoholic liver disease are needed to clarify if PCSK9 inhibitors have the potential to improve alcoholic liver disease.

## 8. PCSK9 and HCV-Induced Chronic Liver Disease

Chronic HCV infections remain a leading cause of liver cirrhosis and HCC [115,116], but direct-acting antivirals (DAAs) that efficiently suppress HCV replication are now available, achieving sustained virologic response rates above 95% [117].

The life cycle of HCV relies heavily on the lipid metabolism of the host as the LDL-R, and probably other lipoprotein receptors such as the VLDL-R or scavenger receptor B1 (SR-B1) (see below), are used by the virus to enter cells. Furthermore, apolipoproteins B, E, AI, AII, CI, CII and CIII contribute to viral replication or production of viral particles [118]. Although it is not fully clarified how LDL-R facilitates HCV entry into cells, an inhibitory role for PCSK9 in HCV infection and propagation was proposed. Indeed, PCSK9 overexpression or supplementation with recombinant PCSK9 reduced LDL-R protein levels and rendered hepatocellular cell lines resistant to HCV infection [119,120]. In support of this, a gain-of-function PCSK9 mutant (D374Y), but not the loss-of-function R194A PCSK9 variant, effectively protected against HCV infection [121].

These findings raised the question if therapeutic inhibition of PCSK9 with evolocumab or alirocumab could increase HCV infection rates. Although this has not yet been evaluated in clinical studies, alirocumab at least did not enhance the entry of HCV particles into the human hepatoma Huh7 cell line [122]. In contrast to models proposing LDL-R to facilitate HCV cell entry, HCV infectivity was slightly higher when alirocumab was used to inhibit PCSK9 in cells lacking the LDL-R. It remains to be determined if these findings are relevant for pathophysiology and HCV entry via other receptors, including SR-B1 [123]. Indeed, only the lack of both LDL-R and SR-B1 receptors efficiently blocked HCV entry, suggesting that interventions targeting only the LDL-R may not significantly alter HCV infectivity [123].

**Figure 2:**
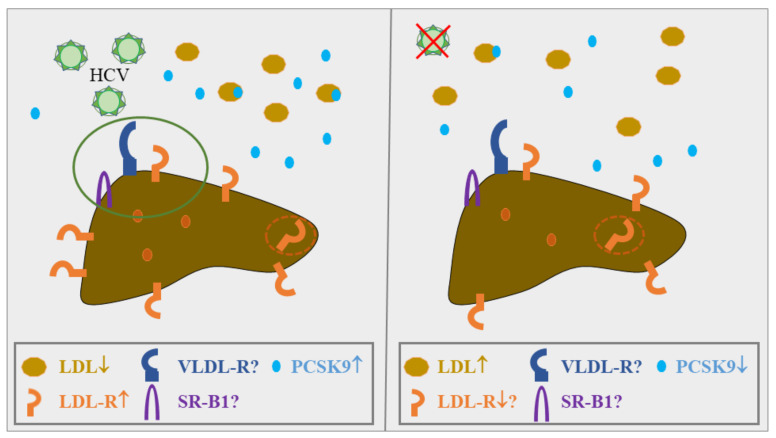
Regulation of LDL, LDL-R and PCSK9 in HCV. HCV infection is associated with high hepatic LDL-R levels and low amounts of circulating LDL, while hepatic and circulating PCSK9 levels are upregulated [31,124,125,127,129]. HCV infection of cells occurs via the LDL-R, the VLDL-R and SR-B1 [118] (encircled in green) and thus, PCSK9 blockage and the associated upregulation of the LDL-R and the VLDL-R was proposed to increase HCV infection [137]. Whether VLDL-R and SR-B1 are also high in the HCV-infected liver has yet to be clarified. Moreover, LDL-R levels were determined by immunoblotting of whole-liver extracts, which cannot discriminate between cell surface-exposed and intracellular LDL-R protein (orange circle with broken lines). However, the decline of LDL argues for a higher functionality of the hepatic LDL-R. Efficient elimination of HCV rapidly increased circulating LDL, whereas PCSK9 levels most likely decline [126,128,129,131,132,133,134,135,136]. Hepatic expression levels of LDL-R, PCSK9, VLDL-R and SR-B1 after viral eradication are still unknown.

Despite these limitations to developing LDL-R as a target to block HCV infection, it is well-established that lipid abnormalities typical for chronic HCV infection, such as hepatic steatosis and hypocholesterolemia [118], would implicate high hepatic LDL-R levels, and accordingly, low PCSK9 levels in chronic HCV. In fact, HCV infection increased LDL-R protein levels in Huh7 hepatoma cells, which correlated with elevated LDL-R expression in the liver of a small cohort of chronic HCV patients [124] (Figure 2). LDL-R protein levels were also increased in the livers of another small cohort of HCV patients in comparison to patients with nonviral liver disease [31] (Figure 2). Although elevated hepatic LDL-R levels in HCV-infected patients need to be validated in larger cohorts [31,124], these results coincide with reduced PCSK9 levels in HCV-infected Huh7 hepatoma cells [124]. However, a clearer picture still has to emerge, as PCSK9 levels were strongly induced in the liver of five HCV patients when compared to five patients with nonviral liver injury [31] (Figure 2).

In line with HCV upregulating hepatic PCSK9 expression, plasma PCSK9 levels were increased in HCV-infected patients compared to control cohorts [31,125,126]. Six different genotypes of HCV exist, and in another study, patients with the major HCV genotype 1 displayed the highest PCSK9 levels, followed by noninfected controls and patients carrying the second most common HCV genotype 3 [127]. These findings implied HCV genotype-dependent effects on plasma PCSK9 levels, but while other studies confirmed HCV-induced PCSK9 serum levels, a link to HCV genotypes was not described [126,127,128,129]. HCV infection also elevated serum PCSK9 levels in noncirrhosis and cirrhosis patients as well as HIV-infected patients [129,130]. On the other hand, LDL levels did not correlate with plasma PCSK9 concentrations in chronic HCV [125,127,129]. Taken together, HCV infection is associated with high PCSK9 and low circulating LDL levels, suggesting that the functional relationship between the LDL-R and PCSK9 is disturbed (Figure 2).

Increased systemic PCSK9 levels were identified in patients with higher viral load [125,127,129]. HCV genotype-related associations may exist between plasma PCSK9 levels and the amount of HCV associated with lipoproteins in ‘lipoviral’ particles (which may also resemble infectious HCV particles) in genotype 3, but not genotype 1 infected patients [127]. Based on current knowledge, HCV infection appears to induce systemic PCSK9 levels by enhancing PCSK9 amounts in the liver. As viral load is a predictor for disease outcome, serum PCSK9 levels may emerge as a prognostic biomarker.

DAA therapy, which efficiently compromises HCV replication, caused a rapid rise of LDL levels in patients with a sustained virologic response in various studies [128,131,132,133,134,135,136] (Figure 2). It remains unclear if DAA-mediated viral elimination alters PCSK9 levels in circulation. One study showed a decline of active PCSK9 levels in serum, and after 4 weeks of DAA therapy, an increase in the less active PCSK9 form. At 28 weeks post-treatment, active PCSK9 was higher, whereas the less active PCSK9 isoform was comparable to pre-treatment concentrations in serum. As active PCSK9 levels were ~5-fold higher compared to the less active variant at the end of the testing period (28 weeks post-treatment), it appears that total PCSK9 in serum declined 4 weeks after commencement of therapy only to steadily increase thereafter [128] (Figure 2). Most studies used commercially available ELISAs to determine systemic PCSK9 levels, and could not discriminate between the active and less active isoforms. In contrast to the abovementioned report, total serum PCSK9 concentrations declined 4 weeks after therapy initiation and remained low up to 12 weeks post-treatment [129]. Reduced circulating PCSK9 levels were also detected in a cohort of 48 HCV-infected patients after successful DAA therapy [126] (Figure 2), while post-treatment plasma PCSK9 concentrations were induced in 27 HCV patients who achieved sustained virologic response [121]. Yet, pretreatment plasma PCSK9 levels did not differ between responders and nonresponders, arguing against an association between systemic PCSK9 levels and efficiency of DAA therapy [121].

PCSK9 is highly expressed in the liver [16], and stage of liver fibrosis may be a confounding factor influencing PCSK9 levels. Noninvasive evaluation of liver fibrosis is recommended and several methods and scores for fibrosis assessment exist. Acoustic radiation force impulse (ARFI), which is based on ultrasound technology, evaluates liver stiffness. Scores such as aspartate aminotransferase (AST)-to-platelet ratio index (APRI) and the fibrosis-4 (FIB-4) score, which is calculated from age, platelet count, AST and alanine aminotransferase (ALT) levels, are also used in clinical practice [138,139,140]. All of these noninvasive methods can accurately exclude and prove advanced liver cirrhosis, but are not reliable for diagnosis of intermediate fibrosis stages [141].

In HCV-infected patients’ serum PCSK9 levels declined with increasing fibrosis stages [129]. Negative correlations of serum PCSK9 concentrations and the MELD score were identified in the cirrhosis group [125,129]. Patients with liver cirrhosis had lower PCSK9 levels than noncirrhosis patients [125,129]. Upon viral eradication, neither PCSK9 nor LDL levels in serum changed in cirrhosis patients [129], identifying an association of liver cirrhosis with reduced serum PCSK9 levels that did not further decline when the virus was eliminated by DAAs [129]. A further study could not identify that serum PCSK9 amounts were reduced in patients diagnosed with liver cirrhosis based on noninvasive tests [126]. Notably, statin use was associated with lower serum PCSK9 levels in this cohort [126].

In summary, HCV infection upregulates circulating PCSK9 concentrations due to enhanced hepatic PCSK9 synthesis and secretion. Circulating PCSK9 levels seem to normalize after efficient eradication of the virus. Whether serum PCSK9 concentrations represent a suitable clinical marker for viral infectivity or success of therapies needs further study.

## 9. PCSK9 and NAFLD Pathogenesis

NAFLD, and its more severe form, NASH, represent the most common chronic liver diseases worldwide. Obesity, insulin resistance and type 2 diabetes are risk factors for NAFLD, for which no treatment is yet available. Patients with NAFLD often exhibit dyslipidemia and hepatic triglyceride and cholesterol accumulation [48,79,142]. Excess of hepatic free cholesterol was also reported in the liver of NAFLD patients, and is considered a cytotoxic lipid with inflammatory and profibrotic effects [143].

**Figure 3:**
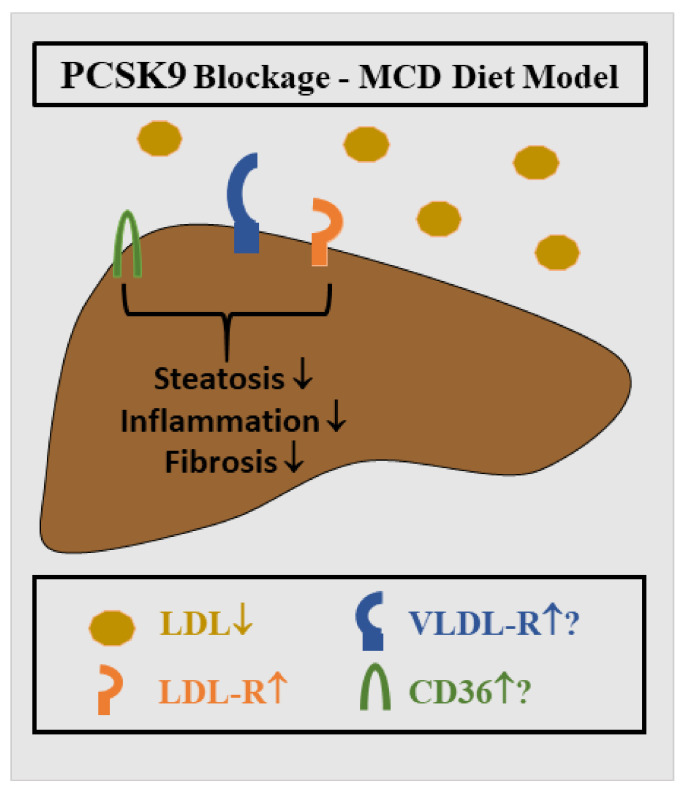
Role of PCSK9 in MCD diet-induced NASH. PCSK9 blockage in mice is associated with low circulating LDL levels and higher LDL-R protein. VLDL-R and CD36 expression in the liver may be also induced. Liver steatosis, inflammation and fibrosis all improved [144].

There is increasing evidence that PCSK9 contributes to NAFLD pathogenesis. Male C57BL/6N mice overexpressing PCSK9 and fed a high-fat diet for 21 weeks had increased plasma cholesterol and triglyceride levels. In these animals, plasma ALT levels, hepatic steatosis, macrophage infiltration and fibrosis scores were increased compared to control animals [29]. In another study, inhibition of PCSK9 upon administration of alirocumab improved murine NASH. In male mice fed a methionine-choline-deficient diet for 6 weeks, which results in hepatic steatosis, oxidative stress, inflammation and fibrosis [144], alirocumab therapy led to reduced numbers of inflammatory cells and lower fibrosis stage, indicating that PCSK9 inhibition may serve as a potential therapeutic approach for NASH (Figure 3). It is important to note that mice fed the MCD diet lose body weight. Here, liver steatosis is caused by impaired export of VLDL particles and increased adipose tissue lipolysis [145,146,147].

**Figure 4:**
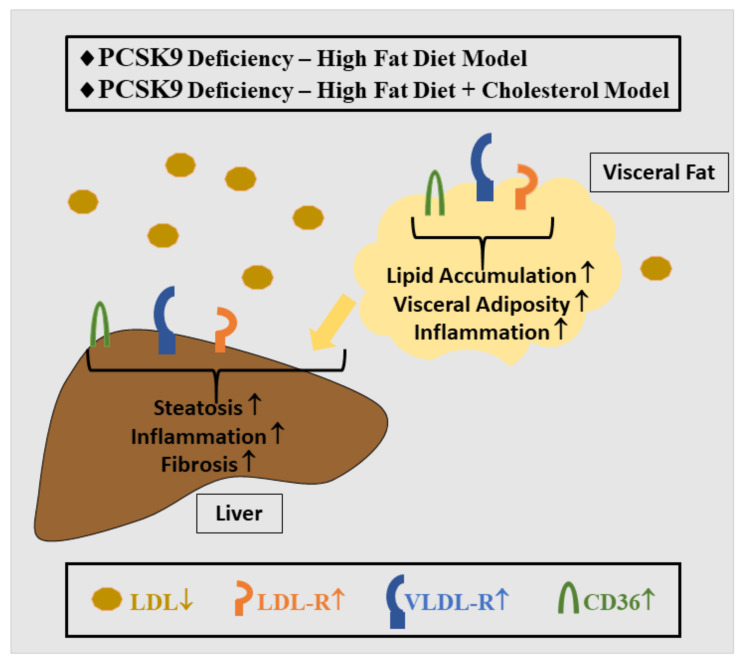
Role of PCSK9 in experimental models of NAFLD. PCSK9 deficiency in mice is associated with low circulating LDL levels and higher LDL-R, VLDL-R and CD36 expression in the liver and visceral fat depots. Separate studies reported PCSK9 deficiency to worsen liver injury. Visceral adiposity was observed in some studies [29,137,144,148,149,150]. Of note, a similar phenotype was also described in mice overexpressing PCSK9 [29]. Whether aberrantly high or low levels of PCSK9 cause a similar phenotype in the NAFLD model will be resolved in future studies.

In contrast to these studies, PCSK9 has also been reported to protect against NAFLD. Feeding male PCSK9 KO mice a high-fat diet for 6 weeks worsened hepatic steatosis, inflammation and fibrosis [137] (Figure 4). Hepatic cholesterol concentrations were comparable in control and PCSK9 KO mice, excluding elevated cholesterol levels in PCSK9-deficient animals to contribute to liver injury. Interestingly, fecal cholesterol was elevated in mice lacking PCSK9, suggesting that low plasma cholesterol levels are related to reduced intestinal cholesterol absorption and/or increased biliary excretion [137]. A further study where PCSK9 null mice were fed a high-fat, high-cholesterol diet nevertheless described accumulation of free cholesterol in the liver. Hepatic inflammation and fibrosis were markedly higher in the mutant mice [148] (Figure 4). Yet, the identification of pathways to explain NASH pathogenesis in PCSK9 null mice needs further study.

Adenoviral overexpression of PCSK9 in mice on a standard diet elevated plasma LDL-cholesterol levels and reduced hepatic LDL-R protein expression, mimicking the phenotype observed in LDL-R-deficient mice, but had no effect on hepatic free cholesterol and cholesteryl ester levels. In bile, neither cholesterol nor phospholipid or bile acid concentrations were affected by increased hepatic PCSK9 expression upon adenoviral infection [4]. While these findings suggest a role for PCSK9 in intestinal cholesterol absorption rather than biliary cholesterol excretion, these data do not support free cholesterol or bile acid accumulation in the liver to cause hepatic damage in the PCSK9 null mice.

Supporting roles for PCSK9 beyond LDL-R downregulation, not only LDL-R, but also VLDL-R and CD36 protein levels were strongly induced in the liver of PCSK9 null mice [137,151] (Figure 4). The fatty acid translocase CD36 mediates fatty acid uptake in many metabolically active tissues and in the liver, and promotes triglyceride accumulation and subsequent lipid-induced endoplasmic reticulum (ER) stress [152]. In PCSK9-depleted HepG2 hepatocarcinoma cells, CD36 mediated the uptake of long-chain fatty acids [137]. Conversely, recombinant PCSK9 or PCSK9 overexpression induced the degradation of CD36 [151], supporting PCSK9 to regulate hepatic CD36 levels. CD36 contributes to hepatosteatosis, and its expression is upregulated in the liver of patients with NAFLD. However, the contribution of CD36 expression in NAFLD progression and NASH is less clear [152].

**Figure 5:**
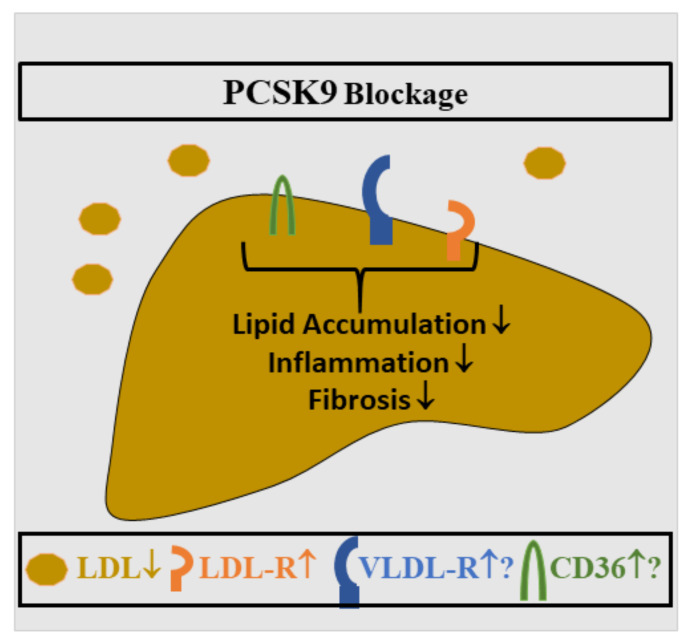
Role of PCSK9 in human liver steatosis. Therapy of severely hyperlipidemic patients with PCSK9 antibodies consistently improved liver steatosis, and a decline in liver inflammation and fibrosis was also observed. Circulating LDL levels were reduced in individuals treated with PCSK9 antibodies, suggesting higher expression of the hepatic LDL-R [153,154,155]. Whether this is associated with increased VLDL-R and CD36 levels needs further analysis.

There is clinical evidence that therapeutic PCSK9 inhibition improves NAFLD. In a cohort of 29 patients, ALT levels and radiologically diagnosed liver steatosis improved in 8 out of 11 patients after PCSK9 antibody treatment for ~2 years. The majority of this patient cohort did not tolerate statins, and conventional therapy could not efficiently reduce lipids in six cohort members [153]. Likewise, in 13 patients with heterozygous familial hyperlipidemia and NAFLD, inhibition of PCSK9 improved hepatic steatosis, inflammation and fibrosis [154]. Furthermore, treatment with PCSK9 inhibitors in a cohort of 26 patients with familial hypercholesterolemia, where statins and ezetimibe did not efficiently reduce LDL cholesterol, improved hepatic steatosis in those patients with a low triglyceride/HDL ratio [155]. Taken together, these studies strongly support PCSK9 blockage to improve hepatic steatosis in patients with severe dyslipidemia (Figure 5).

However, whether loss-of-function PCSK9 mutant carriers are also protected from liver steatosis is less certain. Approximately 2.6% of Europeans are carriers of the PCSK9 loss-of-function variant R46L. In NAFLD patients, this variant protected against more severe hepatic steatosis and fibrosis [29]. In contrast, another study reported 64% R46L variant carriers to display hepatic steatosis, an incidence almost 2-fold higher compared to noncarriers [149]. PCSK9 missense mutant R46L, L253F and A443T carriers all exhibited low LDL levels, while hepatic triglycerides were normal [156]. Individuals with the loss-of-function PCSK9 Q152H variant had normal liver function [157]. Based on these results, it appears that PCSK9 genotypes modify NAFLD pathogenesis only in some patients. One can speculate that this may be related to genetic variations in other genes that modulate PCSK9 function, including Anx A2 (see above), or in genes such as the patatin-like phospholipase domain-containing 3 (PNPLA3) gene, which is well-described to progress NASH [44,158]. Besides the PCSK9 genotype and other contributing gene variations, fat distribution, obesity, insulin sensitivity and gender may be further confounding factors.

Current data on the hepatic expression and circulating levels of PCSK9 in NAFLD are still inconclusive. Hepatic PCSK9 mRNA expression was modestly increased with higher steatosis grade in patients with NAFLD [29]. However, hepatic PCSK9 mRNA levels did not correlate with histological or biochemical markers of disease severity in patients at risk for NASH [159]. Despite the positive correlation of hepatic PCSK9 and fatty acid synthase mRNA expression in morbidly obese patients, a similar association between these two genes in relation to steatosis stage was not observed and hepatic PCSK9 protein levels even declined with increased severity of liver steatosis [160].

Circulating PCSK9 levels were elevated in patients with histologically proven liver steatosis [66], and a positive correlation with the hepatic expression of genes involved in de novo lipogenesis, necroinflammation and fibrosis stages were identified [66]. Notably, plasma PCSK9 levels in healthy controls were strongly associated with clinical markers of liver function, and positively correlated with albumin, alkaline phosphatase, ALT and gamma-glutamyl transferase. This study also observed that plasma PCSK9 concentrations were related to liver steatosis, as judged by ultrasound [161]. In patients at risk for NASH, circulating amounts of PCSK9 correlated with LDL-cholesterol and triglyceride levels, but were not related to histological or biochemical markers of NAFLD [159].

Despite the substantial numbers of clinical and preclinical studies listed above, a firm conclusion on the role of PCSK9 in NAFLD is still lacking. The aforementioned experimental and human data to support a hepatoprotective role of PCSK9 blockage in NASH exist, yet several other (pre-) clinical studies do not support these findings. Although circulating PCSK9 levels are most likely upregulated in patients with NAFLD [161], it appears not suitable as a biomarker for NAFLD in different patient cohorts. Of note, blockage of PCSK9 in dyslipidemic patients improved hepatic steatosis and may prevent NASH [154]. The rather expensive PCSK9 therapy is not recommended in normolipidemic patients, and its impact on liver disease is still unknown. At present, considering the still inconclusive findings from human and experimental models, and lack of knowledge on the patient subgroups that might respond best, it appears unlikely that blockage of PCSK9 will develop into a widespread tool to treat NASH.

## 10. PCSK9 and Other Etiologies of Liver Diseases

Despite chronic HBV infection often being associated with dyslipidemia, the number of studies analyzing PCSK9 in HBV-infected patients is still limited [162]. LDL-R and SREBP expression and activity are both induced upon HBV infection [162] and recent studies also showed LDL-R depletion to inhibit HBV infection in HepG2 cells [163], indicating roles for PCSK9 in this disease setting. In support of these observations, preliminary analysis suggests that PCSK9 protein expression is highly increased in the liver of HBV-infected patients [31]. Accordingly, a case report of a patient with HBV-related HCC and substantial type III hyperlipoproteinemia described high serum PCSK9 levels with PCSK9 mRNA levels being 4-fold higher in the tumor than in nontumor tissues [164]. Further studies are clearly needed to validate if PCSK9 inhibition or LDL-R downregulation can ameliorate dyslipidemia in HBV-infected patients.

## 11. PCSK9 and HCC Progression

The incidence of HCC is constantly rising, and the disease is difficult to treat. Like other cancers, HCC is characterized by a high demand for energy, which is commonly covered by an increased uptake and fermentation of glucose to lactate, also known as the Warburg effect [165]. In addition, there is accumulating evidence that lipid metabolism is altered in liver tumors. This includes an accumulation of triglycerides and cholesterol, while proapoptotic ceramides are reduced [56]. Moreover, there is a shift from unsaturated to saturated lipids, which protects HCC tumors from oxidative stress [56]. Despite plentiful examples of altered cholesterol and lipid homeostasis in oncogenic settings, little is yet known regarding if PCSK9 up- or down-regulation contributes to HCC progression. A recent study described an antiapoptotic and proliferation-promoting role of PCSK9 in HCC cells and an orthotopic human xenograft model [166]. In these studies, PCSK9 amounts correlated with increased tumor size as PCSK9 overexpression increased, while PCSK9 depletion decreased tumor size [166]. An underlying mechanism could be the ability of PCSK9 to upregulate the expression of fatty acid synthase, the latter being overexpressed in many cancers [56,166,167]. In line with PCSK9 expression levels determining tumor size in xenograft models, datasets from human HCC tissues revealed high PCSK9 expression to correlate with microvascular invasion and large tumor size. Accordingly, disease-free survival and overall survival were improved in patients with low PCSK9 expression levels [166].

In contrast, PCSK9 null mice injected with the carcinogen diethylnitrosamine more likely developed HCC than the wild type controls [148].

On the other hand, PCSK9 deficiency, and consequently, increased hepatic clearance of LDL, reduced melanoma metastasis in the liver of mouse models [168]. This decreased metastatic spread was lost upon feeding with a high-cholesterol diet, overriding genetic alterations that lower the risk for cancer cell spreading [168]. Another report showed PCSK9 to interact with glutathione S-transferase Pi 1 (GSTP1), thereby blocking activation of Jun N-terminal kinase (JNK) and consequently inhibiting HCC growth [169]. Furthermore, HCC patients exhibiting low PCSK9 levels had a shorter overall survival and recurrence-free survival time compared to HCC patients with high amounts of PCSK9 [20,169]. These opposite findings reflect data available from the Human Protein Atlas, which based on expression data from 365 patients, classified PCSK9 levels as not prognostic in liver cancer (www.proteinatlas.org, accessed on 16 January 2022) [170].

Nevertheless, tumor and nontumor tissues seem to differentially regulate PCSK9 expression. In patients, PCSK9 mRNA and protein were lower in HCC tissues compared to adjacent tissues [20,169]. These findings coincided with high LDL-R mRNA and protein levels in HCC tissues compared to adjacent nontumor tissues [20]. This might indicate that PCSK9 downregulation in HCC would allow increased LDL-R expression and LDL uptake, features also observed in other tumors and associated with malignant progression [171,172,173]. On the other hand, PCSK9 protein levels in the tumors were not linked to tumor grade [20] and in another case report, PCSK9 mRNA levels were even 4-fold higher in the tumor than in the adjacent tissues [164].

Despite a trend for PCSK9 downregulation in HCC observed in the studies described above, serum PCSK9 levels of HCC patients were comparable to controls, but higher compared to patients with liver cirrhosis or chronic hepatitis. Because only six controls were included in this study, further research is needed to clarify if serum PCSK9 levels in HCC patients differ from healthy individuals [20]. Taken together, current data suggest that PCSK9 levels are reduced in HCC tissues and the association of tumor PCSK9 expression with prognosis needs evaluation in future studies. In consideration of the strong correlation of serum PCSK9 levels with residual liver function, it appears unlikely that circulating PCSK9 concentrations could become a valuable biomarker for HCC diagnosis.

## 12. Expression and Role of PCSK9 in Adipose Tissues

Adipose tissue is an endocrine organ, and has a fundamental role in lipid metabolism, constantly communicating with the liver [79,174]. De novo cholesterol synthesis only plays a minor part in adipocytes, and these cells obtain cholesterol predominantly through endocytosis of lipoproteins via receptors [175], including the LDL-R, which is expressed in adipocytes and facilitates LDL internalization and degradation [174]. The ability of adipocytes to store substantial amounts of cholesterol as cholesteryl esters together with triglycerides further highlights their central role in whole-body lipid homeostasis. Importantly, cholesterol storage in adipocytes is intimately linked to glucose homeostasis, as depletion of cellular cholesterol using cyclodextrins impaired insulin response of adipocytes [175].

Most relevant for this review, PCSK9 not only induces LDL-R degradation in the liver but also in non-hepatic tissues, implicating a contribution to cholesterol homeostasis in adipose tissue (Figure 4) [176]. Along these lines, low plasma PCSK9 levels correlated with increased expression of LDL-R, CD36 and VLDL-R in white adipose tissues [176]. LDL exposure of human adipocytes initially upregulated PCSK9, LDL-R, and SREBP expression. Prolonged LDL incubation led to reduced LDL-R protein levels, suggesting that initial elevation of PCSK9 secretion increased LDL-R degradation at later time points [177]. Treatment of 3T3-L1 adipocytes and murine adipocytes with recombinant PCSK9 lowered LDL-R as well as CD36 protein levels, the latter strongly reducing the internalization of fatty acids [151]. These findings may have far-reaching consequences, as dietary fatty acids are mostly taken up by adipocytes, limiting fatty acid deposition in lean organs such as the liver. High circulating PCSK9 levels may thus impair CD36-mediated uptake of lipids in fat tissues and the liver [151]. This is unlikely to protect the liver from steatosis, as impaired fat storage in adipocytes would increase circulating lipids and ultimately their storage in other organs, with fatty acids entering the liver by alternative pathways. In support of this, long-chain fatty acid uptake in CD36 null mice decreased by 30–40% in adipose tissue, while hepatic long-chain fatty acid levels were comparable in CD36-deficient and wild type mice. Taken together, PCSK9-induced degradation of CD36 appears most important for fat storage in adipose tissues [178].

However, PCSK9-dependent functions for CD36 in hepatocytes seem to exist, as CD36 expression was induced in the liver and perigonadal fat of PCSK9 null mice. This was associated with hepatic accumulation of triglycerides, illustrating that lipid uptake of adipocytes was not sufficient to protect animals lacking PCSK9 from increased hepatic deposition of ectopic lipid [151].

PCSK9 KO mice had normal body weight but accumulated intra-abdominal fat [149,150]. On closer inspection, only perigonadal and perirenal fat mass, but not subcutaneous fat, were induced in PCSK9-deficient animals, a phenotype irrespective of sex, age and diet [149,150]. Fat mass gain in perigonadal and perirenal fat tissues of these animals was due to adipocyte hypertrophy and did not involve the LDL-R, but was likely due to elevated VLDL-R levels, which were markedly induced in visceral fat and normalized upon restoration of hepatic expression of PCSK9 in the PCSK9 null mice [150].

It should be noted that a link between PCSK9 and visceral adiposity was not assessed or even noticeable in several of the experimental models described. For instance, the weight of gonadal, inguinal and brown fat was comparable in male wild type and PCSK9 null animals when fed a normal chow or high-fat diet [137].

The adipose-tissue-related findings obtained from PCSK9 KO animals only partially relate to phenotypes observed in human PCSK9 mutant carriers. For instance, patients with the loss-of-function PCSK9 R46L variant had higher body mass index and more android fat compared to controls [149]. Hence, although PCSK9 inactivation can establish low LDL levels, PCSK9 loss-of-function mutations might concomitantly induce adipocyte hypertrophy in visceral fat depots in a subgroup of patients [33,150], thereby creating a risk factor for insulin resistance, type 2 diabetes and NAFLD [79]. Notably, 64% of carriers of the PCSK9 R46L variant also displayed hepatic steatosis, which reflected a 2-fold higher incidence compared to non-mutant carriers [149].

There is increasing evidence that the cardioprotective action of cholesterol-lowering drugs and common genetic variations associated with low LDL-cholesterol levels lead to an increased risk for type 2 diabetes [179,180]. This includes statins, which are well described to increase the risk for type 2 diabetes [176]. Incidence of type 2 diabetes was also higher in carriers of the loss-of-function PCSK9 R46L variant, which, as outlined above, is associated with low LDL-cholesterol levels [149]. On the other hand, in a cohort of 27 overweight/obese patients with normal serum cholesterol levels, higher plasma PCSK9 concentrations were not linked to LDL, but higher HDL, reduced triglycerides and apolipoprotein B levels [181]. Implicating PCSK9 to regulate LDL-R and CD36 levels in adipocytes of obese patients, increased LDL-R and CD36 expression in white adipose tissue of obese patients with low PCSK9 levels was identified, which correlated with an activation of the NLRP3 inflammasome, higher grade of systemic inflammation and a higher risk for type 2 diabetes [181].

Insights in the underlying mechanisms that may trigger inflammatory events in adipocytes upon LDL uptake are still limited. Differentiation of adipocytes in the presence of LDL lowered lipid storage capacity of mature adipocytes. Together with the downregulation of genes with roles in adipocyte lipid metabolism, this indicates a capacity of LDL to impair adipogenesis. In line with this, these adipocytes produced less adiponectin, while the proinflammatory CCL2 chemokine was induced [181]. Other studies reported that LDL induced adiponectin secretion from primary adipocytes. As lovastatin lowered adiponectin amounts secreted from 3T3-L1 adipocytes, this may indicate that internalized LDL cholesterol modulates critical adipocyte functions. Yet, LDL apheresis, which effectively, but only transiently, lowers LDL levels, did not affect circulating amounts of adiponectin. This argues against an acute effect of LDL elimination on serum adiponectin levels at least in these patients [182].

Taken together, despite the growing literature that hypocholesterolemic drugs and common mutations associated with low LDL cholesterol lead to an increased risk for type 2 diabetes [179,180], the lowering of risk for type 2 diabetes via drugs blocking LDL uptake in fat cells is currently not recommended.

## 13. Conclusions

Inhibition of PCSK9 has become an additional option to reduce cardiovascular risk in patients with high LDL-cholesterol levels. As outlined above, there is some evidence that blockage of PCSK9 may also protect from liver injury. Statin therapy can decrease the incidence of liver cirrhosis [58], and considering the inconsistent reports currently available, further studies are needed to evaluate whether pharmacological PCSK9 inhibition could be advantageous in this regard. PCSK9 blockage has also been linked to visceral adiposity, insulin resistance and a higher risk for type 2 diabetes, all of which contribute to NAFLD. However, more studies are still needed to better understand the impact of PCSK9 blockage in patients with metabolic liver diseases. In addition, PCSK9 may have a role in HCV infection, with several reports suggesting that therapeutic PCSK9 inhibition increases the risk of HCV infection. Positive associations of circulating PCSK9 levels with viral load and rapid decline of PCSK9 concentrations after virus elimination in noncirrhotic HCV patients may initiate studies to clarify whether systemic PCSK9 levels could become a valuable biomarker for the quantification of infectious HCV particles. While still little is known on the role of PCSK9 in HBV, PCSK9 expression is suppressed in HCC tissues, and use of PCSK9-inhibitory antibodies may thus contribute to the development of liver cancers. This has to be evaluated in future studies.

Taken together, cell and animal models and patient cohorts relevant for chronic liver diseases and treated with pharmacological PCSK9 inhibitors have revealed multiple layers of additional PCSK9 functions that extend well beyond its therapeutic aspect in overcoming hypercholesterolemia. Given that disturbed cholesterol metabolism is often associated with different etiologies linked to chronic liver dysfunction, including obesity, insulin resistance, type 2 diabetes, NAFLD, as well as viral infections and liver cancers, it will be important to clarify if PCSK9 inhibition counteracts or contributes to pathophysiology in chronic liver diseases.
